# Trajectories of psychological recovery during inpatient treatment for mood disorders: a longitudinal study of resilience and related psychological resources

**DOI:** 10.3389/fpsyg.2026.1762414

**Published:** 2026-04-10

**Authors:** Kei Sato, Leo Gotoh, Ayuko Yukutake, Michihiko Matsushita, Yuichiro Tokunaga, Tatsuya Ishitake

**Affiliations:** 1 Department of Environmental Medicine, Kurume University School of Medicine, Kurume University, Kurume, Fukuoka, Japan; 2 Shiranui Hospital, Ōmuta, Fukuoka, Japan

**Keywords:** longitudinal study, mood disorders, psychological recovery, resilience, self-compassion

## Abstract

**Background:**

Recovery from mood disorders is often evaluated primarily in terms of reductions in depressive symptoms. However, recovery is also thought to involve psychological changes, particularly in psychological resources such as resilience. The present study aimed to examine longitudinal changes in resilience and related psychological resources—self-compassion and loneliness—alongside depressive symptoms among psychiatric inpatients with mood disorders, and to explore the dynamics of psychological recovery during hospitalization.

**Methods:**

Participants were patients with mood disorders (ICD-10 F3) admitted to a stress-care psychiatric ward in a collaborating hospital. Self-report psychological scales assessing depressive symptoms (SDS, BDI), loneliness (UCLA Loneliness Scale – short form), self-compassion (Self-Compassion Scale – short form), and resilience (RS) were administered at admission and discharge. Changes in psychological measures were calculated, and improvement patterns were examined based on score changes and predefined criteria.

**Results:**

A total of 53 participants were included in the analysis. Significant improvements were observed in all psychological measures between admission and discharge. Improvements in depressive symptoms were observed in more than 90% of participants, whereas improvements in resilience were observed in approximately 70%. Changes in depressive symptoms were significantly associated with changes in resilience, self-compassion, and loneliness, although the associations with loneliness were relatively weaker. Changes in resilience were also positively associated with changes in self-compassion and loneliness.

**Conclusion:**

These findings suggest that improvements in depressive symptoms and improvements in psychological resources, including resilience, self-compassion, and loneliness, do not necessarily occur in parallel during the recovery process. The results highlight potential limitations of evaluating recovery solely based on symptom reduction and suggest the importance of considering multidimensional indicators of recovery that include psychological and social functioning.

## Introduction

Mood disorders represent a major global health challenge requiring urgent attention (World Health Organization, 2017). Depression, the most common mood disorder, carries a high risk of chronicity, and its onset is considered multifactorial. Initial episodes are often triggered by life stressors or adverse events; however, with repeated recurrences, contributing factors have been reported to shift toward internal vulnerabilities (Scher et al., 2005). Furthermore, the onset of mood disorders has been associated with declines in cognitive functioning (Elgersma et al., 2015). Thus, individuals with mood disorders are exposed to an elevated risk of recurrence due to both persistent external stressors and increasing internal vulnerability following onset.

In addressing these risks and promoting recovery, increasing attention has been directed toward psychological capacities related to resistance to illness, such as cognitive functioning and resilience. Resilience has been conceptualized as a construct contrasting with vulnerability and is considered an important psychological characteristic even in crisis situations such as mental illness (Kalisch et al., 2017). Previous studies have debated whether resilience should be conceptualized as a relatively stable trait or as a dynamic process that changes through interactions between individuals and their environments (Southwick et al., 2014). More specifically, previous studies have suggested that temperament and character traits may contribute to individual differences in resilience among patients with affective disorder (Tsigkaropoulou et al., 2024). Previous research has suggested that resilience may include both relatively stable trait components and more dynamic state components that fluctuate depending on environmental conditions and stress exposure (Kalisch et al., 2017; Southwick et al., 2014). Regardless of these conceptual differences, resilience is widely recognized as an important factor supporting psychological adaptation and recovery.

In addition to resilience, other psychological constructs have been recognized as important in the recovery process from mood disorders. Self-compassion refers to the tendency to respond to one’s own suffering with understanding and kindness rather than with self-criticism, and higher levels of self-compassion have been associated with lower depressive symptoms and better emotional regulation. Loneliness, defined as the subjective perception of inadequate or unsatisfactory social connections, has also been linked to depression and psychological distress. These psychological resources may interact with each other and influence psychological functioning during recovery, and like resilience, they have been associated with depressive symptoms and psychosocial adaptation (Hawkley and Cacioppo, 2010; MacBeth and Gumley, 2012).

Although theoretical and clinical discussions have long suggested that symptom remission does not necessarily indicate full psychological recovery, empirical evidence describing how depressive symptoms and psychological resources change together over time remains limited. Previous studies have examined psychological resources related to depression, including resilience and self-compassion, and these constructs have been associated with depressive symptoms and psychological functioning. However, these factors have often been investigated separately, and relatively few studies have longitudinally assessed multiple psychological resources simultaneously within the same clinical population during the recovery process. Recent studies have begun to examine psychological recovery trajectories in patients with mood disorders during treatment; however, empirical evidence remains limited. For example, previous longitudinal research has suggested that treatment environments and psychosocial interventions may influence changes in resilience during treatment among individuals with mood and anxiety disorders (Meule et al., 2023).

In clinical practice, recovery from mood disorders is often evaluated primarily in terms of symptom reduction. However, improvements in depressive symptoms do not necessarily coincide with improvements in psychological resources related to adaptation and psychosocial functioning (Zimmerman et al., 2006). Understanding how these psychological resources evolve during the recovery process may therefore provide important insights into the dynamics and quality of recovery beyond symptom remission. Psychological resources such as resilience, self-compassion, and loneliness have been associated with depressive symptoms and broader mental health outcomes (IsHak et al., 2011). In particular, the extent to which changes in depressive symptoms correspond with changes in different psychological resources during recovery remains insufficiently understood.

The potential influence of comprehensive inpatient interventions and therapeutic environmental factors has been noted in previous research, and studies conducted in stress-care psychiatric wards in Japan have also reported improvements in resilience among patients (Sato et al., 2023). For example, cognitive-behavioral approaches may influence self-compassion by promoting more adaptive self-evaluations, while peer interactions within the inpatient setting may affect patients’ perceptions of social connectedness and loneliness. These processes may also be related to changes in broader psychological resources such as resilience.

At the same time, the variability in individual trajectories suggests that these influences do not operate uniformly across patients. Such heterogeneity may reflect differences in underlying personality traits, cognitive styles, and social factors, which are not fully captured by symptom-based measures alone.

The present study aimed to examine longitudinal changes in depressive symptoms and multiple psychological resources—resilience, self-compassion, and loneliness—during psychiatric hospitalization, and to explore the relationships among these psychological changes.

## Materials and methods

### Participants

Data were collected at Shiranui Hospital, a private psychiatric hospital located in Fukuoka Prefecture, Japan, which has 178 beds including a stress-care ward specializing in the treatment of mood disorders such as depression. In addition to standard psychotherapy and pharmacotherapy, various non-pharmacological interventions are routinely provided in this ward.

Stress-care psychiatric wards in Japan have been established since the 1990’s. These wards primarily admit patients with stress-related conditions such as mood disorders and adjustment disorders. Although the term does not have a strict formal definition, these wards typically provide a therapeutic environment that combines psychosocial interventions, structured daily activities, carefully designed therapeutic settings, and supportive inpatient care. Historically, these wards were established as an additional form of inpatient care within psychiatric services, particularly to accommodate patients with stress-related conditions.

Eligible participants were inpatients newly admitted to the stress-care ward who met the following criteria: (1) diagnosis of a mood disorder (ICD-10: F3), and (2) age between 18 and 65 years.

Diagnoses were made by attending psychiatrists according to ICD-10 criteria as part of routine clinical practice.

### Study period and procedure

Participants were enrolled between 1 October 2023 and 31 December 2024. Within 1 week of admission, all eligible patients received written and verbal explanations of the study, and written informed consent was obtained.

Psychological assessments were conducted at two time points: at admission (baseline) and at discharge (between 3 days prior to discharge and the day of discharge). Demographic characteristics and clinical information were extracted from medical records.

### Psychological assessments

Psychological functioning was assessed using the following self-report measures. Loneliness and self-compassion were included because they are sometimes conceptualized as components or correlates of resilience, allowing for a more comprehensive evaluation of recovery processes.

### Depression

Self-Rating Depression Scale (SDS), Japanese version: a 20-item self-report scale assessing depressive symptom severity. Higher scores indicate more severe depression.

Beck Depression Inventory (BDI), Japanese version: a 21-item self-report measure of depressive symptoms, with higher scores indicating greater severity.

### Psychological measures

Loneliness: The Japanese short version (10 items) of the UCLA Loneliness Scale. This scale conceptualizes loneliness as a unidimensional emotional experience and avoids explicit use of the term “loneliness.” Higher scores indicate greater loneliness and are associated with poorer psychological outcomes Arimoto and Tadaka, 2019).

Self-compassion: The Japanese short version (12 items) of the Self-Compassion Scale, which assesses three positive components (self-kindness, common humanity, and mindfulness) and three negative components (self-judgment, isolation, and over-identification). Higher scores reflect greater self-compassion (Arimitsu et al., 2011).

Resilience: The Resilience Scale (RS), a 25-item self-report measure assessing resilience in terms of individual competence and acceptance of self and life. Higher scores indicate greater resilience (Wagnild and Young, 1993).

### Clinical and treatment variables

Basic clinical information included age, sex, diagnosis, treatment history, employment status, and details of the current hospitalization.

### Pharmacotherapy

Pharmacotherapy during hospitalization was recorded as present or absent.

### Non-pharmacological programs

Although not included in the primary statistical analyses, participation in physician-directed non-pharmacological programs was recorded for descriptive purposes. These included counseling, occupational therapy, mindfulness-based group sessions, Ayurveda-based interventions, and a return-to-work support program. If a program was implemented at least once, it was classified as “introduced.”

### Data handling

In this study, only participants with complete psychological assessment data at both admission and discharge were included in the analyses.

Age categories (high vs. low) used in descriptive tables were defined based on the median age of the sample.

### Statistical analysis

All statistical analyses were conducted using JMP Pro 17 (SAS Institute), with a two-tailed significance level of 0.05.

Changes in psychological measures between admission and discharge were examined using paired *t*-tests. Within-group effect sizes (Cohen’s *d*_*z*_ for paired samples) were calculated using the formula t/√n.

Change scores were calculated as discharge minus admission.

Pearson correlation coefficients were calculated to examine the relationships among changes in depressive symptoms and psychological resources during hospitalization and to explore whether improvements in these measures occurred in parallel.

In addition to analyses of mean score changes, improvement patterns were descriptively examined. Improvement was defined as a favorable change between admission and discharge. For SDS, BDI, and loneliness, improvement was defined as a negative change score (discharge minus admission < 0). For resilience and self-compassion, improvement was defined as a positive change score (discharge minus admission > 0). The number and proportion of participants showing improvement in each measure were calculated.

Multiple linear regression analyses were conducted using the enter method to explore factors associated with changes in psychological resources. Change scores for resilience, self-compassion, and loneliness were used as dependent variables. Independent variables included age, sex, baseline values of each psychological measure, and change in depressive symptoms (SDS) to account for symptom improvement during treatment.

Given the modest sample size, these regression analyses were considered exploratory. No formal correction for multiplicity was applied.

### Ethical considerations

This study was conducted in accordance with the Declaration of Helsinki and the Ethical Guidelines for Medical Research Involving Human Subjects. Ethical approval was obtained from the Ethics Committee of Kurume University and the Ethics Committee of the collaborating research institution.

## Results

### Basic characteristics and inpatient treatment

Table 1 summarizes participants’ baseline characteristics and inpatient pharmacotherapy and non-pharmacological programs. Of the 102 patients who received an explanation of the study, 65 provided consent, and 53 participants with complete psychological assessment data at both admission and discharge were included in the main analyses (51% of those approached).

**TABLE 1:** Baseline characteristics of patients at admission.

Variable	Total (*n* = 53)	Men	Women
Age, years	43.3 (12.23)	44.1	41.9
Diagnosis (ICD-10)			
Bipolar affective disorder (F31)	9 (16.9%)	6	3
Depressive episode (F32)	32 (60.3%)	20	12
Recurrent depressive disorder (F33)	11 (20.7%)	6	5
Persistent affective disorder (F34)	1 (1.8%)	1	0
History of psychiatric treatment			
<3 months	14 (24.5%)	8	5
3 months–1 year	11 (20.7%)	7	4
1–10 years	17 (32.0%)	13	4
>10 years	12 (22.6%)	5	7
Occupation			
Employed person	39 (73.5%)	27	12
Unemployed/housewife	14 (26.4%)	6	8
**Treatment during hospitalization**			
period			
Length of stay, days	64.9 (68.0)	68.8	58.3
Interval between assessments, days	55.7 (59.5)	60.0	48.9
Medication			
Antidepressant use, *n* (%)	33 (62.2%)	23	10
Antipsychotic use, *n* (%)	17 (32.0%)	7	10
Classification of patients by antidepressant and antipsychotic use			
Both	9 (16.9%)	4	5
Antidepressants only	24 (45.2%)	19	5
Antipsychotics only	8 (15.0%)	3	5
Neither	12 (22.6%)	7	5
Additional therapeutic programs			
Counseling	37 (69.8%)	20	17
Occupational therapy	44 (83.0%)	31	13
Mindfulness	18 (33.9%)	8	10
Ayurveda	14 (26.4%)	9	5
Return-to-work support program	12 (22.6%)	12	0

Values are presented as mean (SD) unless otherwise indicated.

The most common diagnosis was depressive episode (F32), and treatment history was broadly distributed. Approximately 70% of participants were employed at baseline.

The mean length of hospitalization was 64.9 days. The mean interval between the first and second psychological assessments was 55.7 days.

Approximately 60% of participants received antidepressants and approximately 30% received antipsychotics during hospitalization; 13 participants (22%) did not receive either antidepressants or antipsychotics.

Non-pharmacological programs were widely implemented, with more than 80% of participants engaging in occupational therapy.

### Changes in psychological measures during hospitalization

Table 2 presents the changes in psychological measures between admission and discharge. Changes were calculated as discharge minus admission. Psychological resources, including resilience, self-compassion, and loneliness, also improved overall.

**TABLE 2:** Comparison of psychological test scores between two time points during hospitalization.

	Admission mean (SD)	Discharge mean (SD)	*P*	Cohen’s *d*_*z*_	Improved *n* (%)
Measure					
SDS	55.0	43.7	<0.001	1.18	49 (92.4%)
BDI	29.8	17.0	<0.001	1.24	51 (96.2%)
Resilience	96.4	106.4	<0.001	0.57	35 (66.1%)
Self-compassion	28.3	32.8	<0.001	0.71	41 (77.3%)
Loneliness	26.1	23.6	<0.001	0.62	43 (81.1%)

Values are mean (SD). *P*-values from paired *t*-tests.

### Distribution of improvement patterns

Most participants showed improvement in depressive symptoms during hospitalization. Improvement was observed in 93% of participants for SDS and 94% for BDI.

In contrast, improvements in psychological resources were less consistent. Resilience improved in 67% of participants, loneliness improved in 81%, and self-compassion improved in 75%.

Importantly, improvements in depressive symptoms and resilience did not always occur concurrently. Among participants who showed improvement in depressive symptoms, 16 individuals (27.5%) did not show a corresponding improvement in resilience.

Table 3 presents correlations among changes in depressive symptoms and psychological resources during hospitalization. Changes in depressive symptoms were significantly associated with changes in resilience and self-compassion, whereas associations with loneliness were weaker. Changes in resilience and self-compassion showed a positive correlation, suggesting that these psychological resources may evolve in parallel during recovery. In contrast, loneliness showed weaker correlations with other psychological indicators.

**TABLE 3:** Correlations among changes in depressive symptoms and psychological resources during hospitalization.

	Change in SDS	Change in BDI	Change in resilience	Change in self-compassion	Change in loneliness
Change in SDS	–	0.75 (<0.001)	−0.52 (<0.001)	−0.53 (<0.001)	0.20 (0.137)
Change in BDI	–	–	−0.55 (<0.001)	−0.64 (<0.001)	0.41 (0.002)
Change in resilience	–	–	–	0.73 (<0.001)	−0.41 (0.002)
Change in self-compassion	–	–	–	–	−0.34 (0.010)

Values represent Pearson correlation coefficients (*r*), with *p*-values in parentheses.

### Multiple regression analyses

Table 4 presents multiple regression analyses with change scores (discharge minus admission) for each psychological measure as dependent variables. Independent variables included age, sex, baseline psychological scores at admission, and change in SDS scores. Given the modest sample size, these regression analyses were considered exploratory.

**TABLE 4:** Multiple regression analyses with changes in psychological test scores (difference between the first and second assessments) as dependent variables.

	Resilience				Self-compassion				Loneliness			
Mean change (standard deviation)	10.0 (17.5)				4.5 (6.3)				−2.5 (4.0)			
	**Estimates**	**Standard error**	**Standardized estimates**	***P***	**Estimates**	**Standard error**	**Standardized estimates**	***P***	**Estimates**	**Standard error**	**Standardized estimates**	***P***
Sex (men)	3.25	2.11	0.18	0.129	0.50	0.82	0.11	0.540	0.07	0.59	0.02	0.900
Age	0.13	0.17	0.07	0.421	0.07	0.06	0.17	0.224	−0.03	0.04	−0.06	0.492
Baseline value of the dependent variable	−0.22	0.09	−0.28	0.028	−0.16	0.13	−0.25	0.234	−0.18	0.09	−0.12	0.050
Change in SDS	−0.86	0.22	−0.47	<0.001	−0.34	0.08	−0.81	<0.001	0.09	0.05	0.14	0.132

Changes in resilience were significantly associated with baseline resilience scores and change in SDS scores. A similar pattern was observed for self-compassion. In contrast, none of the explanatory variables showed significant associations with changes in loneliness.

## Discussion

The present study examined recovery processes in psychiatric inpatients by analyzing changes in psychological assessment scores between admission and discharge. In the present study, depressive symptoms and resilience improved in parallel on average during hospitalization. However, the findings also suggest that, while these constructs are related, they may exhibit partially distinct trajectories during the recovery process (Fava and Ruini, 1996).

On average, depressive symptoms improved substantially during hospitalization. Psychological resources, including resilience, self-compassion, and loneliness, also showed overall improvement. Psychiatric hospitalization may provide a particularly informative context for examining recovery processes, as multiple therapeutic elements—including pharmacotherapy, psychosocial interventions, and intensive therapeutic support—are delivered within a relatively structured environment. In this sense, the inpatient treatment setting may represent a condensed microcosm of the broader recovery process. In the stress-care psychiatric ward examined in this study, pharmacotherapy is typically combined with structured psychosocial programs and a supportive therapeutic environment. The observed improvements may therefore reflect general recovery processes occurring within this inpatient treatment setting.

Research on resilience has expanded rapidly in recent years, with an increasing number of empirical studies examining its relationship with depressive symptoms. Previous studies have reported associations between resilience and depressive symptoms (Meule et al., 2023). However, the extent to which improvements in depressive symptoms and changes in psychological resources occur in parallel during the recovery process remains insufficiently understood.

However, differences emerged when improvement patterns were examined. While depressive symptoms improved in most participants, improvements in psychological resources were less consistent. In particular, a notable proportion of participants showed improvement in depressive symptoms without a corresponding improvement in resilience. These findings suggest that symptom remission alone may not necessarily reflect all aspects of psychological recovery.

In addition, the patterns observed for loneliness and self-compassion appeared more similar to those of resilience than to depressive symptoms. This may suggest that psychological resources follow somewhat different trajectories from symptom reduction during the recovery process. Loneliness was not completely independent from psychological resources, as suggested by its association with resilience (Hawkley and Cacioppo, 2010). These findings may indicate that loneliness represents a social dimension of recovery that partially overlaps with psychological resources while remaining distinct from symptom remission (Hollon et al., 2005).

These discrepancies may not necessarily reflect insufficient treatment effects or limitations of measurement. Rather, they may reflect the possibility that recovery from mood disorders is a multidimensional process. In clinical practice, recovery is often evaluated primarily based on symptom reduction. However, the present findings suggest that improvements in depressive symptoms may occur without corresponding improvements in psychological resources that support longer-term adaptation and functioning.

Even within the relatively intensive treatment environment of the stress-care ward, improvements in resilience were not universal. This may indicate that strengthening psychological resources such as resilience may involve processes that extend beyond short-term symptom improvement. Previous studies have suggested that many patients continue to experience residual psychological vulnerabilities even after outpatient treatment (Liu et al., 2020).

Several limitations should be acknowledged. First, psychological assessments were conducted at only two time points, which limits the ability to draw conclusions about causal relationships. Second, the specific effects of individual therapeutic components could not be examined in detail. Third, the study was not conducted based on *a priori* statistical power calculations. Therefore, the findings should be interpreted with appropriate caution. Given the modest sample size, the regression analyses should be interpreted as exploratory and hypothesis-generating.

The present findings may also be interpreted in light of the multifactorial nature of resilience. Previous studies have suggested that resilience is influenced by a combination of relatively stable personality traits and modifiable cognitive processes. In this context, the overall improvement in resilience observed during hospitalization may reflect the combined effects of multiple therapeutic components, including structured psychosocial interventions, supportive environments, and symptom reduction.

Taken together, these findings suggest the importance of evaluating recovery from mood disorders using multidimensional indicators that extend beyond symptom remission alone. Patients who appear to recover symptomatically may still experience limitations in psychological resources that support long-term psychosocial functioning. Understanding how different psychological resources change during recovery may provide further insight into the complexity of the recovery process and inform future research on treatment and rehabilitation strategies.

## Data Availability

The datasets presented in this article are not readily available due to ethical restrictions. The data contain sensitive clinical information from psychiatric inpatients, and public release could compromise participant privacy and carries a risk of re-identification. Therefore, in accordance with the approved ethical guidelines, the dataset is not available for public access. Requests to access the datasets should be directed to the corresponding author at: Kei Sato, MD, Ph.D. (email: satou_kei@kurume-u.ac.jp).
